# Emergence and maintenance of actionable genetic drivers at medulloblastoma relapse

**DOI:** 10.1093/neuonc/noab178

**Published:** 2021-07-17

**Authors:** Stacey Richardson, Rebecca M Hill, Christopher Kui, Janet C Lindsey, Yura Grabovksa, Claire Keeling, Louise Pease, Matthew Bashton, Stephen Crosier, Maria Vinci, Nicolas André, Dominique Figarella-Branger, Jordan R Hansford, Maria Lastowska, Krzysztof Zakrzewski, Mette Jorgensen, Jessica C Pickles, Michael D Taylor, Stefan M Pfister, Stephen B Wharton, Barry Pizer, Antony Michalski, Abhijit Joshi, Thomas S Jacques, Debbie Hicks, Edward C Schwalbe, Daniel Williamson, Vijay Ramaswamy, Simon Bailey, Steven C Clifford

**Affiliations:** 1 Translational & Clinical Research Institute, Faculty of Medical Sciences, Newcastle University Centre for Cancer, Newcastle upon Tyne, UK; 2 The Hub for Biotechnology in the Built Environment, Department of Applied Sciences, Northumbria University, Newcastle upon Tyne, UK; 3 Department of Applied Sciences, Northumbria University, Newcastle upon Tyne, UK; 4 Department of Onco-Haematology, Cell and Gene Therapy, Bambino Gesù Children’s Hospital—IRCCS, Rome, Italy; 5 Department of Pediatric Hematology and Oncology, AP-HM, Marseille,France; 6 CHU Timone, Service d’Anatomie Pathologique et de Neuropathologie, AP-HM, Marseille, France; 7 Institute of NeuroPhysiopathology, Aix-Marseille Universite, CNRS, Marseille, France; 8 Children’s Cancer Centre, Royal Children’s Hospital, Murdoch Children’s Research Institute, Melbourne, Victoria, Australia; 9 Department of Pathology, The Children’s Memorial Health Institute, Warsaw, Poland; 10 Department of Neurosurgery, Polish Mother’s Memorial Hospital Research Institute, Lodz, Poland; 11 Department of Haematology and Oncology, Great Ormond Street Hospital for Children, London, UK; 12 Department of Histopathology, Great Ormond Street Hospital for Children, London, UK; 13 Developmental Biology and Cancer Research & Teaching Department, UCL Great Ormond Street Institute of Child Health, London, UK; 14 Programme in Developmental and Stem Cell Biology, The Hospital for Sick Children, Toronto, Ontario, Canada; 15 Hopp Children’s Cancer Center Heidelberg (KiTZ), Heidelberg, Germany; 16 Division of Pediatric Neurooncology, German Cancer Research Center (DKFZ), Heidelberg, Germany; 17 Department of Pediatric Hematology and Oncology, Heidelberg University Hospital, Heidelberg, Germany; 18 Sheffield Institute for Translational Neuroscience, University of Sheffield, Sheffield, UK; 19 Oncology Unit, Alder Hey Children’s Hospital, Liverpool, UK; 20 Department of Cellular Pathology, Royal Victoria Infirmary, Newcastle upon Tyne, UK

**Keywords:** drivers, genomics, medulloblastoma, relapse, subgroups

## Abstract

**Background:**

Less than 5% of medulloblastoma (MB) patients survive following failure of contemporary radiation-based therapies. Understanding the molecular drivers of medulloblastoma relapse (rMB) will be essential to improve outcomes. Initial genome-wide investigations have suggested significant genetic divergence of the relapsed disease.

**Methods:**

We undertook large-scale integrated characterization of the molecular features of rMB—molecular subgroup, novel subtypes, copy number variation (CNV), and driver gene mutation. 119 rMBs were assessed in comparison with their paired diagnostic samples (n = 107), alongside an independent reference cohort sampled at diagnosis (n = 282). rMB events were investigated for association with outcome post-relapse in clinically annotated patients (n = 54).

**Results:**

Significant genetic evolution occurred over disease-course; 40% of putative rMB drivers emerged at relapse and differed significantly between molecular subgroups. Non-infant MBSHH displayed significantly more chromosomal CNVs at relapse (*TP53* mutation-associated). Relapsed MB_Group4_ demonstrated the greatest genetic divergence, enriched for targetable (eg, *CDK* amplifications) and novel (eg, *USH2A* mutations) events. Importantly, many hallmark features of MB were stable over time; novel subtypes (>90% of tumors) and established genetic drivers (eg, SHH/WNT/P53 mutations; 60% of rMB events) were maintained from diagnosis. Critically, acquired and maintained rMB events converged on targetable pathways which were significantly enriched at relapse (eg, DNA damage signaling) and specific events (eg, 3p loss) predicted survival post-relapse.

**Conclusions:**

rMB is characterised by the emergence of novel events and pathways, in concert with selective maintenance of established genetic drivers. Together, these define the actionable genetic landscape of rMB and provide a basis for improved clinical management and development of stratified therapeutics, across disease-course.

**Key Points**

1. Genetic events both emerge and are selectively maintained between diagnosis and relapse.
2. The molecular genetics and temporal evolution of rMB are subgroup-specific.
3. rMB events converge on targetable pathways and predict outcome post-relapse.

**Importance of the Study**

Despite the grave prognosis of rMB, biopsy at relapse is uncommon, and molecularly stratified trials at relapse are rare. Only a few modestly sized studies have investigated rMB biology and/or its clinical potential. The largest whole genome sequencing study to date (n = 43) suggested that rMBs are highly genetically divergent from their diagnostic counterparts.

Our large-scale genetic characterization of rMB (n = 119), at the level of disease drivers, challenges prior findings regarding its genetic divergence: many putative genetic drivers at relapse (60%) are maintained from diagnosis and thus actionable across disease-course. Importantly, our study also reveals molecular subgroup-dependent evolution at relapse, identifying novel and targetable genetic events not previously appreciated at diagnosis.

Critically, this extensive genetic characterization of rMB strongly supports routine rMB sampling to guide clinical management post-relapse. Integrated analysis highlights the involvement of emergent biological pathways at relapse, far exceeding their involvement at diagnosis. Moreover, specific genetic events are prognostic post-relapse.

There is urgent unmet need for the development of novel therapeutic strategies for relapsed medulloblastoma (rMB). Following upfront primary therapy, relapse occurs in around one-third of medulloblastoma (MB) patients and is almost universally fatal, accounting for approximately 10% of childhood cancer deaths.^1,2^ However, the genetic landscape of rMB and its potential for clinical exploitation are not well understood. Understanding the genetic drivers of rMB and their relationship to the disease at diagnosis will be essential to underpin future therapeutic advances.

Over the last decade, comprehensive genome-level investigations have led to significant advances in our understanding of the genetic basis of MB at diagnosis. Four consensus molecular subgroups are recognized, each associated with distinct genomic, demographic, and prognostic profiles (MB_WNT_ [favorable-risk]; MB_SHH_, MB_Group3_, and MB_Group4_ [all intermediate-risk]^3^). Furthermore, investigations of over 1000 tumors at diagnosis have identified subgroup-defining mutations (eg, *CTNNB1* in MB_WNT_, SHH pathway genes in MB_SHH_), additional recurrently mutated genes (eg, *DDX3X*, *KMT2C*), and novel molecular subtypes have recently been described.^4–6^ Alongside these, validated prognostic biomarkers have been identified (eg, *TP53* mutation and *MYCN* amplification in MB_SHH_; *MYC* amplification in MB_Group3_ [all high-risk]).^3^ Together, these now form the basis of routine MB diagnostics and risk-adapted therapy stratification in current biomarker-driven clinical trials of upfront therapies (eg, SJMB12, NCT01878617 and SIOP-PNET5-MB, NCT02066220).^3^

In contrast, there have not been equivalent investigations of the relapsed disease, primarily due to a lack of re-sampling; molecularly stratified trials of targeted therapies at relapse are uncommon. Studies of initial rMB cohorts have shown that while molecular subgroup is predominantly stable, other molecular features are commonly altered at disease recurrence.^7–10^ Most recently, Kumar et al showed that the degree and patterns of molecular conservation at relapse vary according to disease subgroup.^11^ Mutations of *TP53* have been commonly identified in rMB, and can be both selectively maintained from diagnosis, or acquired at disease relapse.^8–10^ However, while genetic divergence at relapse has been reported, the role of additional, specific genetic events, including any maintained from diagnosis, remain to be established.

Exploiting rMB biology to improve disease outcomes will require detailed understanding of its genetic landscape, any molecular evolution over disease-course, the extent to which genetic aberrations are acquired or selectively maintained at relapse, and any relationships between disease molecular evolution and upfront therapies received. Beyond characterization of genetic events, consideration of their clinical utility will be essential, in terms of opportunities to (i) identify putative therapeutic targets, (ii) integrate with early-phase clinical trials strategies, and (iii) direct clinical management at both diagnosis and relapse.

Here, we report the assembly and genetic characterization of a cohort of 119 relapsed tumors and consider these alongside (i) matched diagnostic counterparts and (ii) a large independent reference cohort sampled at diagnosis. Using an integrated analysis of these cohorts, we identify the key genetic events, which constitute the genetic landscape of rMB, and assess its evolution over disease-course.

## Methods

### Cohort Assembly

Tumor material was obtained from UK CCLG institutions and collaborating centers at the time of MB relapse (55/57 had matched diagnostic tumor material). DNA methylation array and clinical data were collected for an additional 28 rMBs (matched diagnosis, n = 28). All patients had a documented clinical remission on imaging prior to relapse and survival data, and year of diagnosis was available for 54 patients. 19/54 (35%) patient tumor samples were collected in 2010-2014, 21/54 (39%) in 2000-2009 and 14/54 (26%) 1993-1999. Alongside a pathologic diagnosis of rMB, DNA methylation-based classification was used to confirm MB diagnoses in our tumor cohort (www.molecularneuropathology.org/mnp).^12^ Consequently, 4/85 (4.7%) recurrent tumors with DNA methylation array data were confidently classified as non-MB (2 Ewing sarcoma, 2 glioblastoma) and were excluded. Informed consent was obtained for all subjects, and human tumor investigations were conducted with approval from Newcastle/North Tyneside Research Ethics Committee (study reference 07/Q0905/71). To expand our cohort, next-generation sequencing data for an additional 38 rMBs (molecular subgroup, n = 35; matched diagnostic, n = 28; matched germline, n = 25)^9^ were obtained with authorization from the International Cancer Genome Consortium (ICGC; EGAD00001000946). The total combined cohort thus consisted of 119 rMBs (matched diagnosis, n = 107).

For comparison, we assembled an independent control cohort from a published tumor study using 282 MBs sampled at diagnosis (dMB), with molecular subgroup, age at diagnosis, DNA methylation array, and mutational datasets available.^6^ Further clinical annotation was not available, however, molecular demographics of this cohort are consistent with previous studies of MB at diagnosis. Cohorts are summarized in Supplementary Tables 1 and 2.

### Molecular Subgrouping

Tumors were assigned to consensus molecular subgroup as previously described.^4,13^ Samples with a subgrouping confidence score >0.7 were assigned into MB_WNT_, MB_SHH_, MB_Group3_, MB_Group4_. Molecular subgroup could be assigned for 116 rMBs (subgroup was unavailable for 3 rMBs obtained from ICGC). Patients were further subclassified according to contemporary age-dependent treatment conventions^3^; age-defined molecular subgroup could be assigned for 100 rMBs. MB_SHH-Infant_ patients (<4 years at diagnosis) were strongly associated with receipt of cranio-spinal irradiation (CSI)-sparing upfront treatments (100%; 10/10 MB_SHH-Infant_ with available data received no CSI at diagnosis). Few assessable infant tumors were available for other subgroups (MB_Group3_, n = 4; MB_Group4_, n = 4); therefore, all remaining subgroup analyses were restricted to non-infants (>4 years at diagnosis) for MB_WNT_, MB_SHH Non-infant_, MB_Group3_, and MB_Group4_ (100%; 35/35 with available data received CSI at diagnosis). Second-generation molecular subtypes were assigned as described in the Supplementary Methods.^5^ Where subtype data were available, MB_SHH-Infant_ corresponded to subtypes gamma and beta, while MB_SHH Non-infant_ comprised alpha and delta subtypes (Supplementary Methods).

### Copy Number Analysis

Tumor samples were analyzed for chromosomal arm-level and focal copy number changes (CNVs). Due to the inherent difficulties in distinguishing true novel driver CNV from passenger CNV in our rMB cohort, focal copy number analysis interrogated 63 genes (Supplementary Table 4) previously reported as regions of recurrent somatic CNV in MB at diagnosis (focal regions <12 Mb).^6,14^ Detailed description of copy number analyses are provided in the Supplementary Methods. CNV events were categorized as “Acquired” (detected in relapsed tumor, not detected in matched diagnostic), “Maintained” (detected in relapsed and matched diagnostic), or “Unknown at diagnosis” (detected in relapsed tumor, matched diagnostic tumor material not available or assessable due to data quality).

### Mutational Analysis

Mutational analysis interrogated 71 genes (Supplementary Table 5) previously reported as frequently mutated putative driver genes in diagnostic MB as well as any additional predicted pathogenic mutations acquired between diagnosis and relapse.^6,9,15^ Detailed description of mutation analyses are provided in the Supplementary Methods. Variants were predicted pathogenic (ie, putative driver mutations) if their consequence included coding or splice donor/acceptor mutations, population allele frequency <0.01, and deleterious by CAROL and FATHHM prediction tools.^16,17^ Variants with allele frequency ≥20% and total read depth ≥10 were further curated by visual inspection in Integrative Genomics Viewer (IGV).^18^ Mutation events were categorized as “Acquired,” “Maintained”, and “Unknown at diagnosis” as described for CNV.

### Pathway Analysis

Whole-cohort and subgroup-specific gene set enrichment analyses were used to investigate the enrichment of known canonical pathways within our combined focal CNV and mutational datasets. Using the Molecular signatures database (version 7.1, https://www.gsea-msigdb.org/gsea/msigdb/)^19^ and Gene Set Enrichment Analysis (GSEA) software,^20^ we computed the overlap with curated canonical pathways (BIOCARTA, KEGG, and REACTOME), with a maximum gene set of 300 and FDR-adjusted *q* value <0.05 considered significant.

### Survival Analysis

Survival analyses were based on patients with available clinical and molecular data (Supplementary Table 2). All recurrently detected (ie, n ≥ 3) molecular and clinicopathological rMB features were tested for association with time-to-death post-relapse (Supplementary Table 3). Individual missing data points were assumed to be missing completely at random for all analyses. The log-rank test was used in univariable analyses to assess time to relapse and time from relapse to death, and the Kaplan-Meier method to visualize results. Cox proportional hazards models were used to investigate the significance of all covariates for time-to-death post-relapse in univariable and multivariable models, using forward likelihood ratio testing. Data were censored for patients who died of other causes or were alive with disease. The Benjamini-Hochberg procedure was employed in univariable analyses to control the false discovery rate and adjusted *P* values <.05 identified significant associations. We tested the proportionality assumption for Cox modeling using scaled Schoenfeld residuals. Proportional covariates with an unadjusted *P* value <.1 in univariable analyses were considered as candidates for multivariable modeling. Due to cohort size, multivariable modeling outputs were limited to 2 covariates. Analysis and visualization were performed using the R statistical environment (version 3.5.3).

### Statistical Analysis

Two-tailed Fisher exact and chi-square tests were used to determine nonrandom associations between categorical mutational and CNV variables using IBM SPSS Statistics for Windows (version 25.0) and encompassed patients with available data for covariates tested. Two-tailed Wilcoxon signed rank and Kruskal-Wallis tests were used to determine whether the number of mutational and CNV events were significantly different between matched diagnosis and relapse samples, and across molecular subgroups, using the R statistical environment (version 3.5.3). The significance threshold for all statistical tests was set at *P* < .05.

## Results

### Subgroup and Subtype Conservation Between Diagnosis and Relapse

**Fig. 1:**
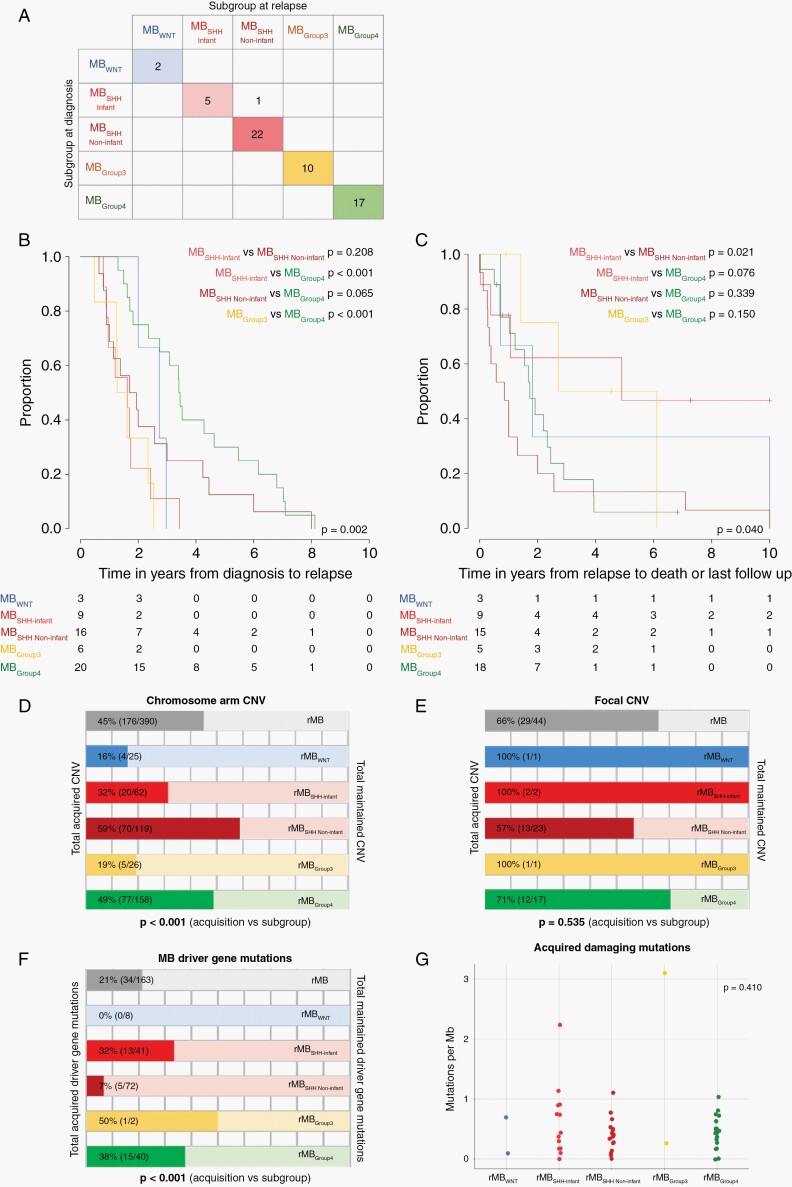
Medulloblastoma subgroups: genetic landscape and disease-course at relapse. (A) Cross-tabulation of MB subgroup at diagnosis and relapse for all matched pairs with confidence score >0.7. (B) Time-to-relapse and (C) time from relapse to death or last follow-up for defined molecular subgroups. *P* values are reported for log-rank tests. (D) Frequency of acquired vs maintained chromosome arm CNVs, (E) focal CNVs, and (F) MB driver gene mutations. *P* values are reported for chi-square tests of association. (G) Total number of damaging mutations per Mb acquired between diagnosis and relapse pairs. *P* values are reported for Kruskal-Wallis test of assessable groups (rMB_SHH-Infant_ (n = 12), rMB_SHH Non-infant_ (n = 16), rMB_Group4_ (n = 19). Abbreviations: CNV, copy number variation; MB, medulloblastoma; Mb, megabase; SHH, sonic hedgehog; WNT, wnt/wingless.

Molecular subgroups and novel molecular subtypes were largely stable over disease-course, with notable exceptions. DNA methylation array data which could be confidently classified (confidence score >0.7) were available at both diagnosis and relapse for 57 patients.^4^ Of these, 56 (98%) maintained consensus molecular subgroup assignment at relapse (Figure 1A). Novel MB_SHH_ subtypes remained stable at relapse in 18/20 (90%) (Supplementary Figure 1).^4,21^ Similarly, considering novel MB_Group3_ and MB_Group4_ subtypes,^5^ 20/24 (83%) pairs maintained their subtype at relapse. Four matched tumor pairs, all subtype VIII at diagnosis, altered subtype at relapse; two of which were further supported by tSNE classification. Three of these 4 patients switched to subtype V at relapse and one to subtype VII. There were no other molecular features recurrently associated with these subtype VIII switches (Supplementary Figure 1).

### Disease-Course in Relapsing Patients Is Associated With Molecular Subgroup and CSI at Relapse

As expected,^2^ time from diagnosis to relapse differed significantly between molecular subgroups in patients with survival data (*P* = .002, log-rank test) (Figure 1B). Molecular subgroup was also associated with disease progression post-relapse (Figure 1C). Most survivors belonged to the MB_SHH-Infant_ subgroup, associated with receipt of radiotherapy at relapse (time-to-death MB_SHH-Infant_ vs MB_SHH Non-infant_, *P* = .021, log-rank test).

### Emergence and Maintenance of Genetic Events at Relapse Differs Between Subgroup

We surveyed the copy number variation (CNV) and mutational landscape (ie, established MB focal copy number events and putative driver mutations) of rMB across all subgroups (Supplementary Figure 2). Significant disease evolution occurred; overall, 40% (239/597) of rMB events emerged at relapse. However, a notable level of conservation was also observed over disease-course; the majority (60%; 358/597) of genetic aberrations detected in rMB was maintained from their matched diagnostic counterpart.

The classes of molecular genetic alteration observed at relapse, and their frequency, differed significantly between the defined subgroups (Figure 1D–F). rMB_Group4_ tumors were most altered at relapse, showing considerable rates of acquisition of all classes of genetic alteration. In contrast, rMB_WNT_ and rMB_Group3_ showed least change, with very few acquired CNVs or mutations. Overall, the total number of additional damaging gene mutations acquired at relapse was equivalent across all assessable subgroups (Figure 1G).

### Infant and Non-Infant MB_SHH_ Have Distinct Genetic Landscapes at Relapse

rMB_SHH-Infant_ and rMB_SHH Non-infant_ differed in the classes and frequencies of genetic alterations acquired. A greater proportion of chromosomal arm-level CNV changes were acquired in rMB_SHH Non-infant_ than rMB_SHH-Infant_ (59%; 70/119 vs 32%; 20/62; *P* < .001, Fisher exact test). However, contrary to expectation, rMB_SHH Non-infant_ (associated with upfront CSI therapy) had far fewer acquisitions of putative driver gene mutations than rMB_SHH-Infant_ (7%; 5/72 vs 32%; 13/41; *P* = .001, Fisher exact test), which was typically treated with radiation-sparing approaches at diagnosis.

SHH pathway mutations were common in both rMB_SHH-Infant_ (eg, *SUFU*, *PTCH1*) and rMB_SHH Non-infant_ (eg, *PTCH1*, *SMO*) and were frequently maintained between diagnosis and relapse (rMB_SHH-Infant_ 6/6; 100% and rMB_SHH Non-infant_ 6/8; 75%, with data available).

**Fig. 2:**
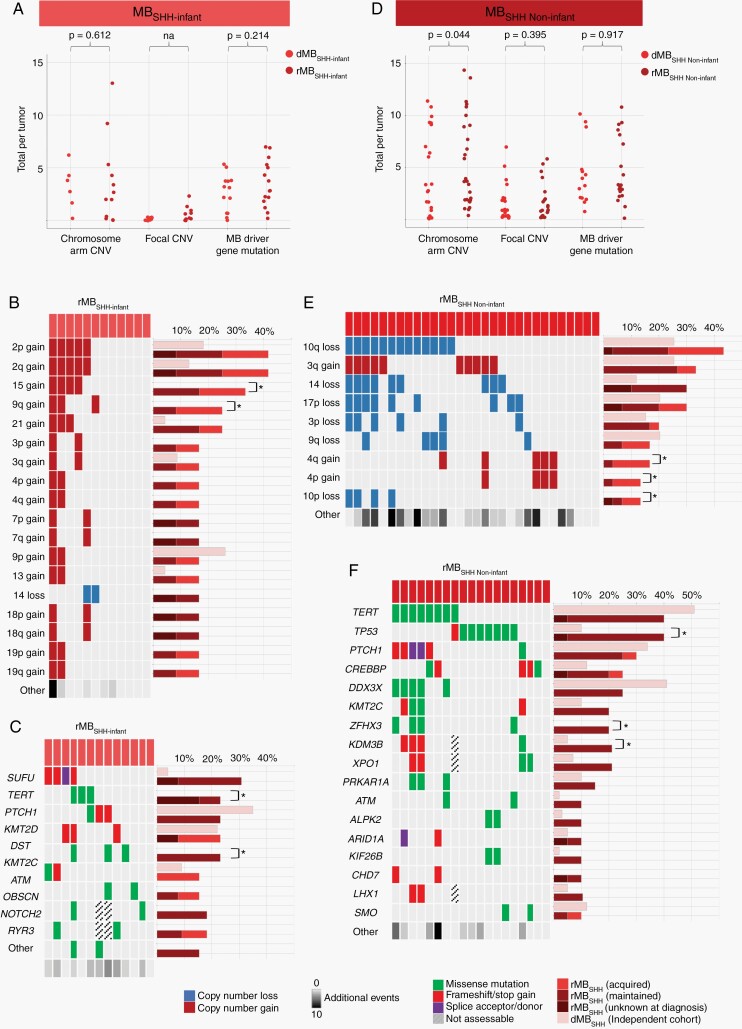
MB_SHH_ in infants and non-infants have distinct genetic landscapes at relapse. (A) Total number of genetic aberrations in matched diagnosis and relapsed MB_SHH-Infant_ tumors. *P* values are represented for Wilcoxon signed rank tests. (B) Frequent chromosome arm CNVs and (C) driver gene mutations in rMB_SHH-Infant_. Bar chart represents the frequency of genetic aberrations in independent dMB_SHH-Infant_ (light pink) and rMB_SHH-Infant_ cohorts. * indicates *P* value <.05, Fisher exact test. (D) Total number of genetic aberrations in matched diagnosis and relapse MB_SHH Non-infant_ tumors. *P* values represented for Wilcoxon signed rank test. (E) Frequent chromosome arm and (F) driver gene mutations in rMB_SHH Non-infant_. Bar chart represents the frequency of genetic aberrations in the independent dMB_SHH Non-infant_ (light pink) and rMB_SHH Non-infant_ cohorts. * indicates *P* value <.05, Fisher exact test. Total number of other genetic aberrations is indicated, with darker shades of gray indicating greater number of events. Copy number gain (dark red), copy number loss (dark blue), missense mutation (green), frameshift/stop gain (red), splice acceptor/donor (purple). Abbreviations: CNV, copy number variation; dMB, diagnostic medulloblastoma; rMB, relapsed medulloblastoma; SHH, sonic hedgehog.

The numbers of putative driver gene mutations and CNVs were not significantly increased between diagnosis and relapse in our matched rMB_SHH-Infant_ tumor cohort (Figure 2A). However, a comparison of our rMB_SHH-Infant_ cohort to a large, independent diagnostic cohort (dMB_SHH-Infant_, n = 23) revealed the enrichment of specific genetic events (Figure 2B and C). Gain of chromosome 15 was enriched by both maintenance and acquisition and, overall, was observed in 33% (4/12) of rMB_SHH-Infant_ vs zero (0/23) in the independent dMB_SHH-Infant_ cohort (*P* < .001, Fisher exact test).

A significantly increased number of chromosomal arm-level CNVs was observed between diagnosis and relapse in our matched rMB_SHH Non-infant_ cohort (Figure 2D, *P* = .044, Wilcoxon signed rank test). Significant enrichment of chromosome 4p/4q gain and 10p loss were observed at relapse when compared to the independent dMB_SHH Non-infant_ cohort, which were predominantly acquired between diagnosis and relapse (Figure 2E, Supplementary Figure 3). Notably, the increased number of chromosomal arm-level CNVs within rMB_SHH Non-infant_ was associated with *TP53*-mutated tumors (*TP53*-mutated [n = 7 tumors], mean 8.14 vs 3.8 *TP53* wild-type [n = 10 tumors], *P* = .032, Mann-Whitney *U* test) (Supplementary Figure 3).

In contrast, no significant increases in the total number of focal CNV and putative driver gene mutation were observed between diagnosis and relapse in our matched rMB_SHH Non-infant_ cohort (Figure 2D). However, a significantly increased number of *TP53* mutations were observed, all maintained from diagnosis (rMB_SHH Non-infant_ 8/19; 42% vs independent dMB_SHH Non-infant_ cohort 6/59; 10%, *P* = .004, Fisher exact test) (Figure 2F). Notably, we did not observe a statistically significant enrichment of *MYCN* amplification (rMB_SHH Non-infant_ 5/28; 18% vs independent dMB_SHH Non-infant_ 5/59; 8%; *P* = .281, Fisher exact test) (Supplementary Figure 3).

### *TP53* Mutations Are Prevalent in rMB_WNT_

**Fig. 3:**
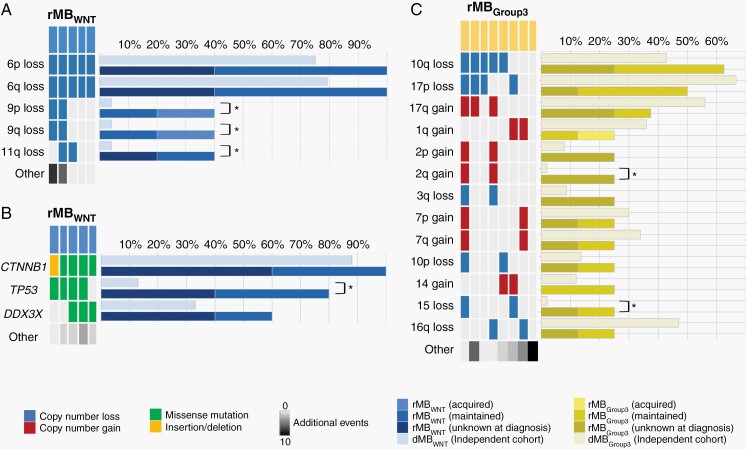
Genetic characteristics of rMB_WNT_ and rMB_Group3_. (A) Frequent chromosome arm CNVs and (B) driver gene mutations in rMB_WNT_. Bar chart represents the frequency of genetic aberrations in independent dMB_WNT_ (light blue) and rMB_WNT_ cohorts. * indicates *P* value <.05, Fisher exact test. Total number of other genetic aberrations is indicated, with darker shades of gray indicating greater number of events. (C) Frequent chromosome arm CNVs in rMB_Group3_. Bar chart represents the frequency of genetic aberrations in independent dMB_Group3_(light yellow) and rMB_Group3_ cohorts. * indicates *P* value <.05, Fisher exact test. Total number of other genetic aberrations is indicated, with darker shades of gray indicating greater number of events. Copy number loss (dark blue), copy number gain (dark red), missense mutation (green), insertion/deletion (gold). Abbreviations: CNV, copy number variation; dMB, diagnostic medulloblastoma; rMB, relapsed medulloblastoma; WNT, wnt/wingless.

As anticipated, based on the excellent prognosis of MB_WNT_ disease at diagnosis (>90% progression-free survival^22^), rMB_WNT_ tumors had limited representation in our rMB cohort (n = 6). As expected, the most frequent genetic aberrations identified in rMB_WNT_ were characteristic monosomy of chromosome 6 and *CTNNB1* mutation, both found in 100% (5/5) of cases and maintained from diagnosis to relapse (Figure 3A and B, Supplementary Figure 3). However, in addition, a number of specific changes emerged or were enriched at relapse. Most notably, mutations of *TP53* were significantly enriched, detected in 4/5 (80%) rMB_WNT_ (vs 3/24 [13%] in the independent dMB_WNT_ cohort [*P* = .007, Fisher exact test]), and maintained from diagnosis were assessable (Figure 3B).

### Recurrent Genetic Events Are Rare in rMB_Group3_

MB_Group3_ tumors were relatively underrepresented in our rMB cohort (n = 10), likely reflecting lack of historical biopsy due to their clear clinical disease-course (more rapid, widely disseminated relapses).^2^ Consistent with MB_Group3_ at diagnosis, relatively few recurrent putative driver mutations and focal CNV events were detected in rMB_Group3_ (data not shown), however, enrichment of chromosome 2q gain and chromosome 15 loss was observed when compared to the independent dMB_Group3_ cohort (Figure 3C, Supplementary Figure 4).

### Emergent Genetic Events Are Most Common in rMBGroup4

**Fig. 4:**
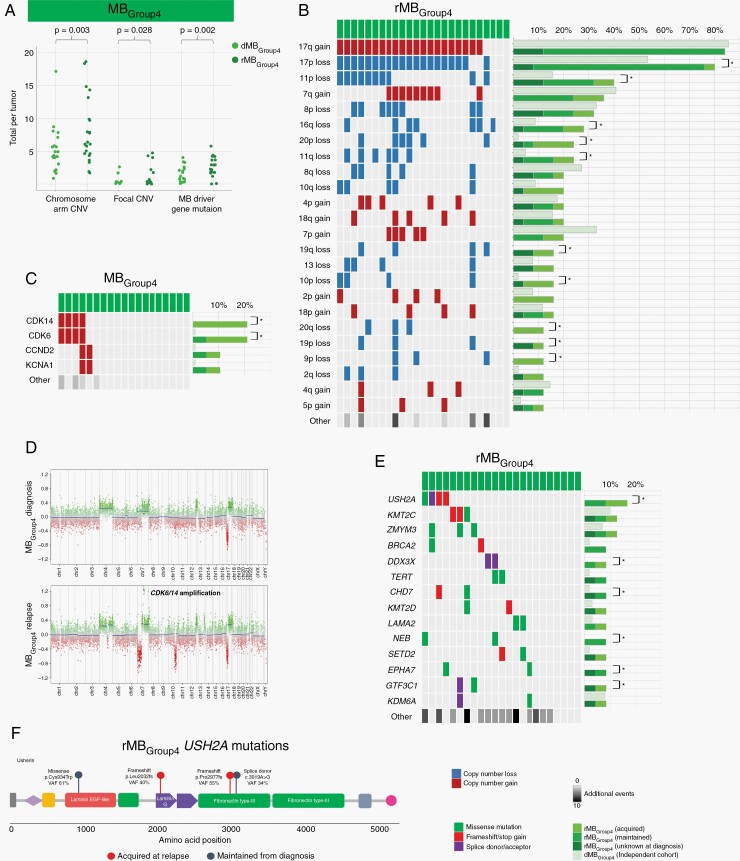
Emergent genetic events are most common in rMB_Group4_. (A) Total number of genetic aberrations in matched diagnosis and relapse MB_Group4_. *P* values represented for Wilcoxon signed rank test. (B) Frequent chromosome arm, (C) focal CNVs, and (E) driver gene mutations in rMB_Group4_. Bar chart represents the frequency of genetic aberrations in independent dMB_Group4_ (light green) and rMB_Group4_ cohorts. * indicates *P* value <.05, Fisher exact test. Total number of other genetic aberrations is indicated, with darker shades of gray indicating greater number of events. Copy number loss (dark blue), copy number gain (dark red), missense mutation (green), frameshift/stop gain (red), splice acceptor/donor (purple). (D) Acquisition of *CDK6/CDK14* amplification in rMB_Group4_. (F) rMB_Group4_*USH2A* mutation type and location on the Usherin protein. Abbreviations: CNV, copy number variation; dMB, diagnostic medulloblastoma; rMB, relapsed medulloblastoma.

Genetic features of rMB_Group4_ differed most markedly from the disease at diagnosis, harboring a significantly increased burden of mutations and CNVs at relapse (Figure 4A, Supplementary Figure 4). At the chromosome arm level, losses of 17p and 11p were enriched in rMB_Group4_ compared to the independent diagnostic reference cohort, observed in 80% (20/25) and 40% (10/25) of rMB_Group4_, respectively, predominantly through maintenance from diagnosis. In contrast, losses of chromosome arms 9p, 10p, 20p, and 20q were significantly enriched, predominantly through acquisition at relapse (Figure 4B, Supplementary Figure 4). While, overall, relatively few focal CNV events were observed in rMB_Group4_, a striking and significant enrichment of *CDK6* and *CDK14* co-amplifications was identified (Figure 4C). These co-amplifications were predominantly acquired at relapse and were present in 21% (4/19) of rMB_Group4_ relapses compared to <1% of the independent dMB_Group4_ (1/103, *P* = .002, Fisher exact test) (Figure 4C and D). Finally, in contrast to a relative paucity of putative driver gene mutations in MB_Group4_ at diagnosis,^6^ we identified several recurrent mutations in rMB_Group4_, a number of which (eg, *USH2A*, *DDX3X*, *CHD7*, *NEB*, *EPHA7*, *GTF3C1*) showed significant enrichment compared to the independent diagnostic reference cohort. Notably, deleterious *USH2A* mutations were most common, identified in 4/23 of rMB_Group4_ (17%; 2 frameshift, 1 missense, 1 splice donor) compared to zero (0/103) occurrences in the independent dMB_Group4_ cohort (*P* = .001, Fisher exact test). These were enriched at relapse by both acquisition (n = 2) and maintenance (n = 2) from diagnosis (Figure 4E and F).

### Biological Pathways Are Enriched at Relapse Through Acquired and Maintained Genetic Events

**Fig. 5:**
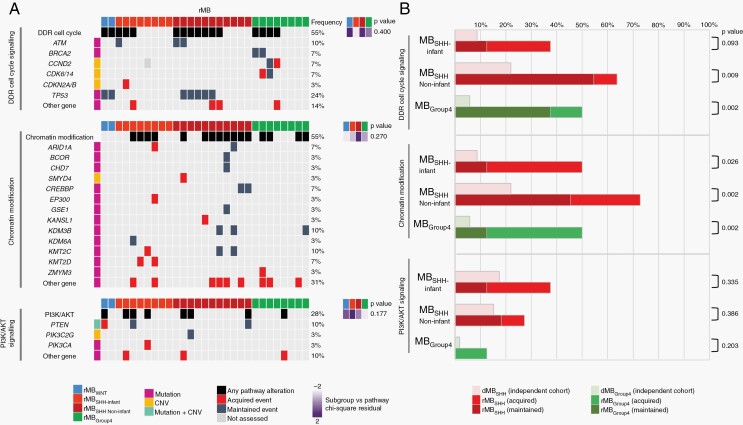
Biological pathways are enriched at relapse through acquired and maintained genetic events. (A) Summary of chromatin modification, PI3K-AKT, and DDR/cell cycle genetic pathway alterations in a restricted matched cohort for which complete mutational/CNV datasets were available at diagnosis and relapse (n = 29 tumors). Each column represents 1 relapsed tumor. Genetic pathway aberration presented (black), acquired aberration (red), maintained aberration (dark gray). *P* values and residual scores from chi-square tests of association are shown alongside with darker shades of purple indicating stronger enrichment. (B) Frequency of combined genetic pathway alterations by molecular subgroup in rMB and an independent dMB cohort, *P* values reported for Fisher exact tests. Abbreviations: CNV, copy number variation; DDR, DNA damage response; dMB, diagnostic medulloblastoma; rMB, relapsed medulloblastoma.

Our interrogation of focal CNV aberrations and mutations identified several low-frequency/singleton events at relapse. We therefore investigated the hypothesis that observed genetic events converge on common critical biological pathways, by undertaking an unbiased approach to investigate enrichment of known canonical pathways within our combined focal CNV and mutational gene sets. Most notably, this analysis identified several key pathways which were significantly enriched in both whole-cohort and subgroup-specific analyses at relapse (Supplementary Figure 5), including chromatin modification, PI3K-AKT signaling, and cell cycle/DNA damage response (DDR) pathways. To investigate these pathways further, we interrogated the nature and frequency of genetic pathway aberrations in a restricted cohort with complete mutational and CNV datasets for both relapse and matched diagnostic pairs (n = 29) (Figure 5A). As previously, we compared the combined frequency of pathway alterations in rMB with the equivalent independent dMB cohort (Figure 5B).

Overall, events associated with DDR/cell cycle signaling were observed in 55% (16/29) of all rMB, greatly exceeding the frequency of mutations observed in *TP53* alone (*TP53*-mutated, 24%; 7/29). In addition to *TP53*, additional recurrent DDR pathway gene aberrations such as *ATM* and *BRCA2* were identified. DDR aberrations were observed across all molecular subgroups at relapse but were significantly enriched in CSI-treated tumors (rMB_SHH Non-infant_, rMB_Group4_), with maintenance from diagnosis to relapse the predominant mode of enrichment. Chromatin-modifying pathway aberrations were observed in 55% (16/29) of all rMB and were significantly enriched in all subgroups assessed, with acquisition between diagnosis and relapse the predominant mode of enrichment. In contrast to DDR pathway aberrations, no single gene dominated, and pathway aberrations were contributed to by a repertoire of low-frequency events (Figure 5A and B). PI3K/AKT signaling pathway aberrations, including both CNV and mutational events, occurred in 28% (8/29) of all rMB, and in all subgroups. While *PTEN* was the most frequently affected gene (*PTEN* CNV/mutation; 3/29 [10%]), the frequency of genetic PI3K/AKT alteration was increased in rMB by a range of singleton gene mutations, most of which were acquired in relapsed tumors.

### Specific rMB Events Predict Disease-Course Post-Relapse

Clinical annotation of our cohort (n = 54; Supplementary Table 2) enabled an initial exploration of whether molecular genetic assessment of MB at relapse has potential to guide clinical management post-relapse. We therefore undertook a cohort-wide analysis of all molecular features observed recurrently at relapse, alongside molecular subgroup and clinical features, to explore any association with disease-course post-relapse (ie, time-to-death).

**Fig. 6:**
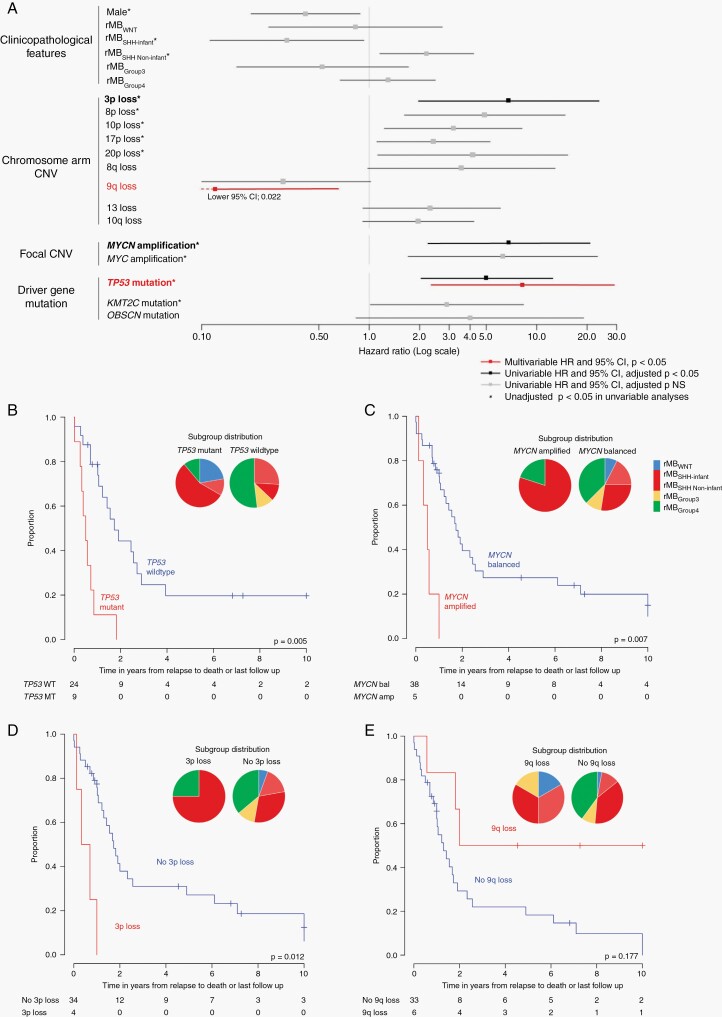
Time from relapse to death is associated with molecular features at relapse. (A) Univariable and multivariable analyses of correlates of time from relapse to death in the clinical cohort. All covariates displayed were entered into multivariable analyses (significant in multivariable analyses displayed in red). (B) *TP53* mutations, (C) *MYCN* amplification, and (D) 3p loss at relapse are associated with more rapid time-to-death. (E) 9q loss at relapse is associated with a prolonged time-to-death (multivariate hazard ratio *P* < .05). Abbreviations: CNV, copy number variation; MB, medulloblastoma; SHH, sonic hedgehog; WNT, wnt/wingless.

Time-to-death post-relapse was molecular subgroup-dependent (Figure 1C). In addition, *TP53* mutation, *MYCN* amplification, and 3p loss were each associated with a more rapid time-to-death in univariable analyses (Figure 6). *TP53* mutation was the most frequent adverse prognostic event observed at relapse (9/36, 25%, Supplementary Table 3); all patients harboring this aberration died within 2-year post-relapse. Notably, clinical features such as disease location and treatment at relapse (chemotherapy, focal radiotherapy/CSI) were not associated with time-to-death post-relapse in univariable analyses (Supplementary Table 3).

Multivariable Cox modeling identified *TP53* mutations (unfavorable) and 9q loss (favorable) as independent risk factors for time-to-death. Overall, 8/20 (40%) of the 4 events associated with disease-course post-relapse (*TP53* mutation, *MYCN* amplification, 3p loss, or 9q loss) were acquired at relapse and not detected at diagnosis, highlighting the importance of biopsy at relapse to guide further clinical management.

## Discussion

Understanding the nature and extent of genetic divergence at medulloblastoma relapse is essential to direct treatment strategies and improve clinical outcomes for this extremely poor prognosis patient group. Our study of 119 rMBs has enabled characterization of the molecular landscape of medulloblastoma relapse, alongside exploration of its potential for clinical exploitation.

Both molecular subgroups and novel subtypes remained stable in the majority (>90%) of relapses. Notably, a small subset (4/16) of MB_Group4_ did switch subtype at relapse. All switchers were subtype VIII tumors at diagnosis but were not associated with other specific molecular characteristics. This is in contrast to findings by Kumar et al, which similarly reported subtype divergence in 15/69 rMB (subtypes III, V, VI, VII, VIII at diagnosis), and proffered an association with *MYC* amplification or chromosome 2p gain at relapse.^11^ However, such cases were infrequent in both studies, and the nature, biological and clinical significance of these rare subtype switchers requires further investigation. Against the background of subgroup stability, emergence and maintenance of putative driver mutations and CNVs were the major mechanisms that shaped the molecular genetic landscape of relapsed medulloblastoma. The rMB landscape differed markedly from the established landscape previously described at MB diagnosis. Overall, the involvement of maintained vs emergent events was comparable to that observed in other relapsed brain tumors (eg, recurrent glioblastoma).^23^

Importantly, around 40% of putative driver mutations and CNVs detected were acquired at relapse. Acquired changes differed significantly in nature between subgroups. Divergent evolution in MB_SHH_ was different between its component subgroups, associated with different upfront therapies and genetic backgrounds. Interestingly, MB_SHH Non-infant_ displayed significantly more chromosomal arm-level CNVs at relapse which were associated with *TP53* mutation. MB_Group4_ was most altered and harbored most genetic events at relapse, in contrast to the paucity of molecular alterations and actionable targets observed in this subgroup at diagnosis.^3^ rMB_Group4_ acquired significant levels of both mutations and CNVs. These included actionable (eg, *CDK6/CDK14*) and enriched (*USH2A*) mutations, not previously identified in disease-wide mutational studies at diagnosis, presenting potential therapeutic opportunities (eg, CDK inhibitors). The discovery of *USH2A* mutations reveals novel and potentially exploitable mechanistic insights into rMB_Group4_, particularly in view of the established role of *USH2A* defects in other diseases (retinitis pigmentosa [OMIM:613809], Usher syndrome [276901]^24^). While the present study sought to identify genomic events which are detectable in bulk tumor profiles, utilization of single-cell and deep sequencing technologies are now required to provide further insight into the origins and evolution of acquired events (eg, clonal evolution vs de novo events).^8^

Like subgroup, 60% of genetic events detected at relapse were maintained from diagnosis, including continued selection of established drivers of MB_WNT_ (*CTNNB1* mutation) and MB_SHH_ (SHH pathway mutations—*PTCH1*, *SUFU*, *SMO*) tumorigenesis. The maintained selection of key pathways over disease-course (eg, SHH/WNT/P53 pathways) supports their relevance at both diagnosis and relapse, and the utility of molecular diagnostics of the tumor at diagnosis to stratify associated targeted therapies (eg, SMO inhibitors for SHH tumors^25^) throughout the disease-course.

Comparison to the independent diagnostic MB cohort identified a number of genomic events which are significantly enriched in our relapsed cohort and therefore highlight potentially critical mechanisms of disease recurrence. The detection of *TP53* mutations in most (4/5) rMB_WNT_ tumors, and their maintenance from diagnosis, contrasts with current understanding that *TP53* mutation status does not carry prognostic value in MB_WNT_,^26,27^ and therefore now requires confirmation in independent cohorts and further investigation.

Several key lines of evidence provide proof-of-principle for the further actionability of our findings. First, rare mutations in single genes converge on a series of critical pathways which are enriched at relapse. The level of involvement of DNA damage repair signaling, chromatin modification, and PI3K signaling (in 55%, 55%, 28% of rMB, respectively) greatly exceeds that of specific individual genes when considered in isolation. This suggests widely relevant opportunities for pathway-directed therapeutic targeting, at both diagnosis and relapse, for further validation and investigation. Second, post-relapse disease-course varies significantly. Specific events were significantly associated with clinical outcome, providing putative biomarkers for stratification of post-relapse disease management, which now require validation in expanded and/or independent cohorts. Finally, we identified a subset of non-MB tumors at apparent disease relapse and biopsy is required for their differential diagnosis.^10,12^ Appropriate preclinical models of rMB are now urgently required to translate genomic studies into mechanistic understanding of MB relapse and improved clinical outcomes.

## Supplementary Material

noab178_suppl_Supplementary_Figures

noab178_suppl_Supplementary_Tables

noab178_suppl_Supplementary_Methods

## Notes

**Authorship statement.** Study design and manuscript writing: S.R., R.M.H., and S.C.C. Provision of tumor samples and clinical information: M.V., N.A., D.F.B., J.R.H., M.L., K.Z., M.J., J.C.P., T.S.J., M.D.T., S.M.P., B.P., and A.Mi. Provision of methylation array data and clinical information: V.R. Collection and processing of tumor tissue: S.C., S.R., R.M.H., and J.C.L. Collection of patient data and clinical interpretation: R.M.H., C.Ke, D.H., and S.B. Pathological review of tumor tissue: S.B.W., A.J., and T.S.J. Laboratory experimentation of tumor cohorts: S.R., R.M.H., and J.C.L. Analysis of tumor cohorts: S.R., R.M.H., C.Ku, J.C.L., Y.G., E.C.S., and D.W. Bioinformatic analysis pipelines: M.B. and L.P. Preparation of figures: S.R. and R.M.H. Contribution to and approval of manuscript: all authors.
