# Supplementary material for: Emergence and maintenance of actionable genetic drivers at medulloblastoma relapse
Source: Neuro Oncol. 2021 Jul 17;24(1):153–65. doi: 10.1093/neuonc/noab178 (PMC8730763; doi:10.1093/neuonc/noab178)
Supplement: noab178_suppl_Supplementary_Figures [file noab178_suppl_supplementary_figures.pdf]

Supplementary Fig. 1

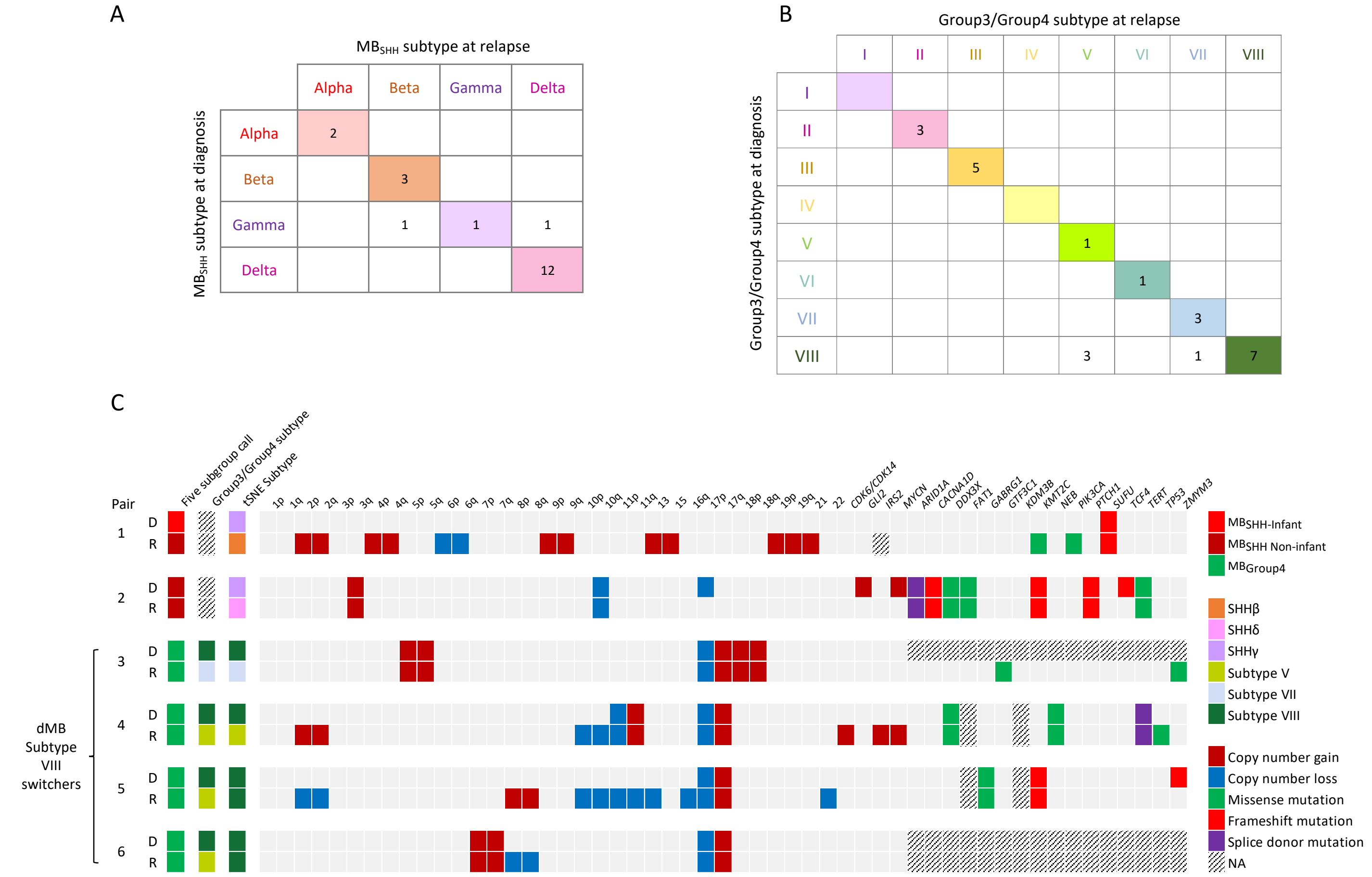

Supplementary Fig. 1. Second generation molecular subgroups/subtypes.
(A) Second generation MB_{SHH} subtypes[1] at diagnosis and relapse for all MB_{SHH} pairs which could be confidently classified by tSNE (ratio of nearest to second nearest cluster <0.7). (B) Second generation MB_{Group3}/MB_{Group4} subtypes[2] at diagnosis and relapse for all matched pairs with DNA-methylation array based classification confidence score > 0.7[3].(C) Genetic characterization of subtype switching patients. D= diagnostic tumor. R= relapse tumor.

1. Cavalli, F.M., et al., Intertumoral heterogeneity within medulloblastoma subgroups. Cancer cell, 2017. 31(6): p. 737-754.
2. Sharma, T., et al., Second-generation molecular subgrouping of medulloblastoma: an international meta-analysis of Group 3 and Group 4 subtypes. Acta neuropathologica, 2019. 138(2): p. 309-326.
3. Capper, D., et al., DNA methylation-based classification of central nervous system tumours. Nature, 2018. 555(7697): p. 469-474.

Supplementary Fig. 2

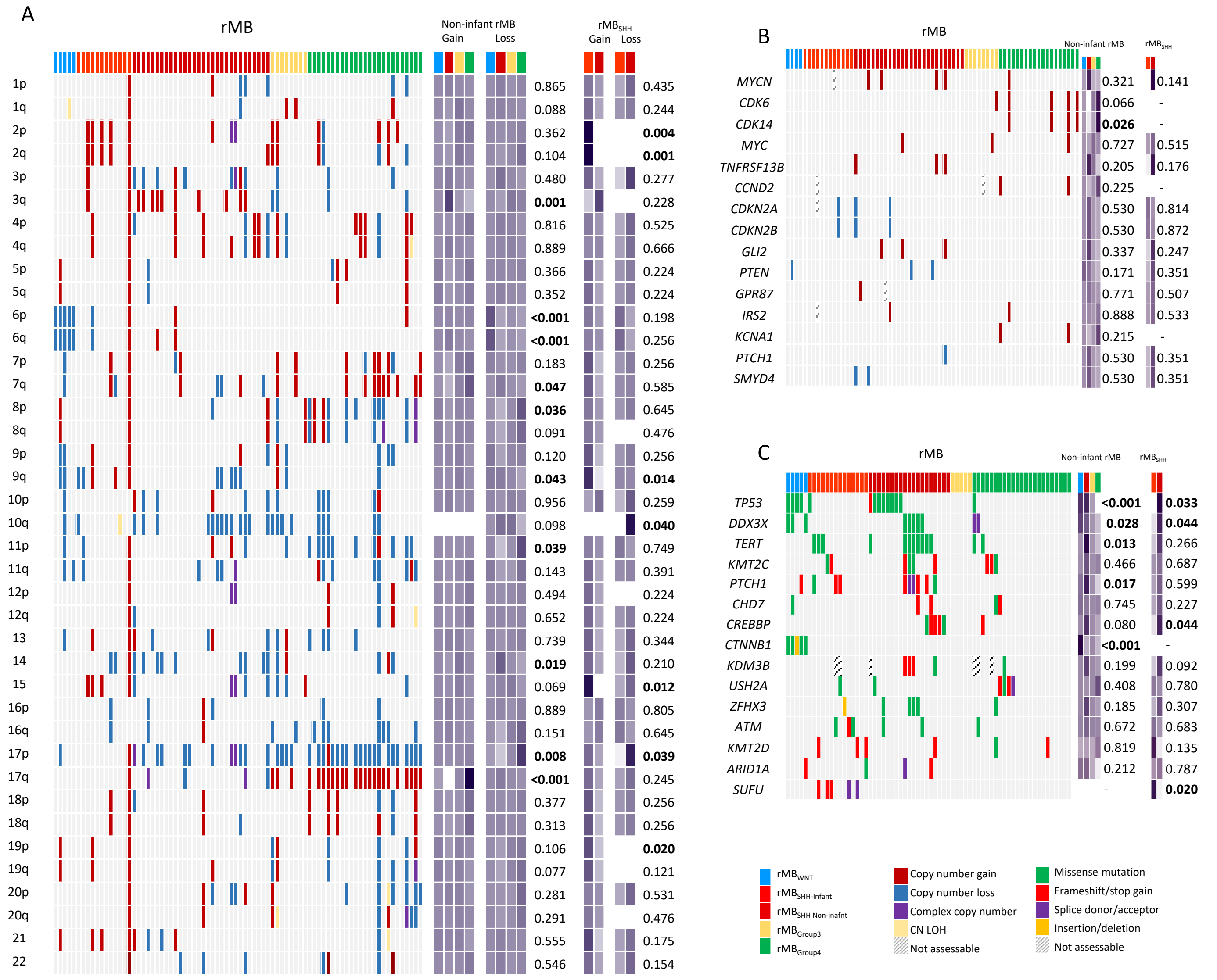

Supplementary Fig. 2. Molecular landscape of rMB.
(A) Chromosome arm CNVs, (B) most frequent focal CNVs. Each column represents one relapsed tumor, copy number gain (dark red), copy number loss (dark blue), complex copy number change (purple), CN LOH (yellow). (C) most frequent driver gene mutations. Each column represents one relapsed tumor, missense mutation (green), frameshift/stop gain (red), splice donor/ acceptor (purple), insertion/deletion (gold). P values and residual scores for two-tailed Chi-square tests of association are shown alongside with darker shades of purple indicate stronger enrichment. CNV = copy number variation. rMB= relapsed medulloblastoma. SHH=sonic hedgehog. WNT=wnt/wingless. CNV=copy number variation. CN LOH = copy number neutral loss of heterozygosity.

Supplementary Fig. 3

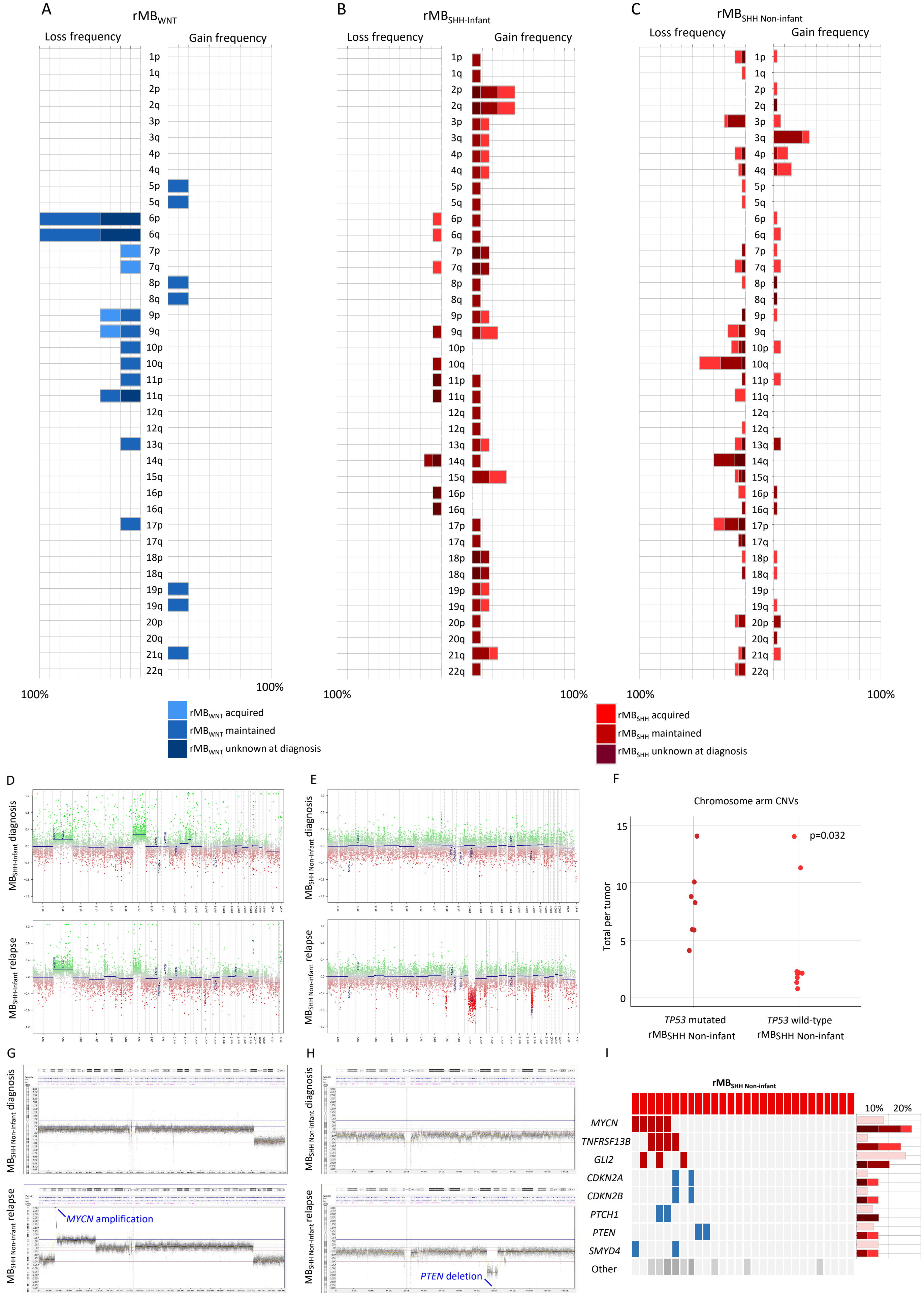

Supplementary Fig. 3. CNV in rMBWNT and rMBSHH

(A) All chromosomal arm level CNVs in rMBWNT (B) All chromosomal arm level CNVs in rMBSHH-Infant. (C) All chromosomal arm level CNVs in rMBSHH Non-infant.(D) Conumee copy number of matched diagnostic and relapse MBSHH-Infant showing maintenance of chromosome 2 gain amongst other CNVs. (E) Conumee copy number of matched diagnostic and relapse MBSHH Non-infant showing acquisition of 10q and 17p loss at relapse. (F) Total number of chromosomal arm level CNVs in *TP53* mutated rMBSHH Non-infant (n=7 tumors) versus *TP53* wild-type rMBSHH Non-infant (n=10 tumors), p=0.032, two-tailed Mann-Whitney U test. (G) Acquisition of focal *MYCN* amplification at relapse in rMBSHH Non-infant shown in Nexus. (H) Acquisition of focal *PTEN* deletion at relapse in rMBSHH Non-infant shown in Nexus. (I) Frequent focal CNV in rMBSHH Non-infant. Bar chart represents frequency of aberrations in independent dMBSHH Non-infant (light pink) and rMBSHH Non-infant. Number of additional focal CNV aberrations is indicated, with darker shades of grey indicating greater number of CNVs. Copy number gain (dark red), copy number loss (dark blue). dMB= diagnostic medulloblastoma. rMB=relapsed medulloblastoma. CNV=copy number variation. WNT=wnt/wingless. SHH=sonic hedgehog.

Supplementary Fig. 4

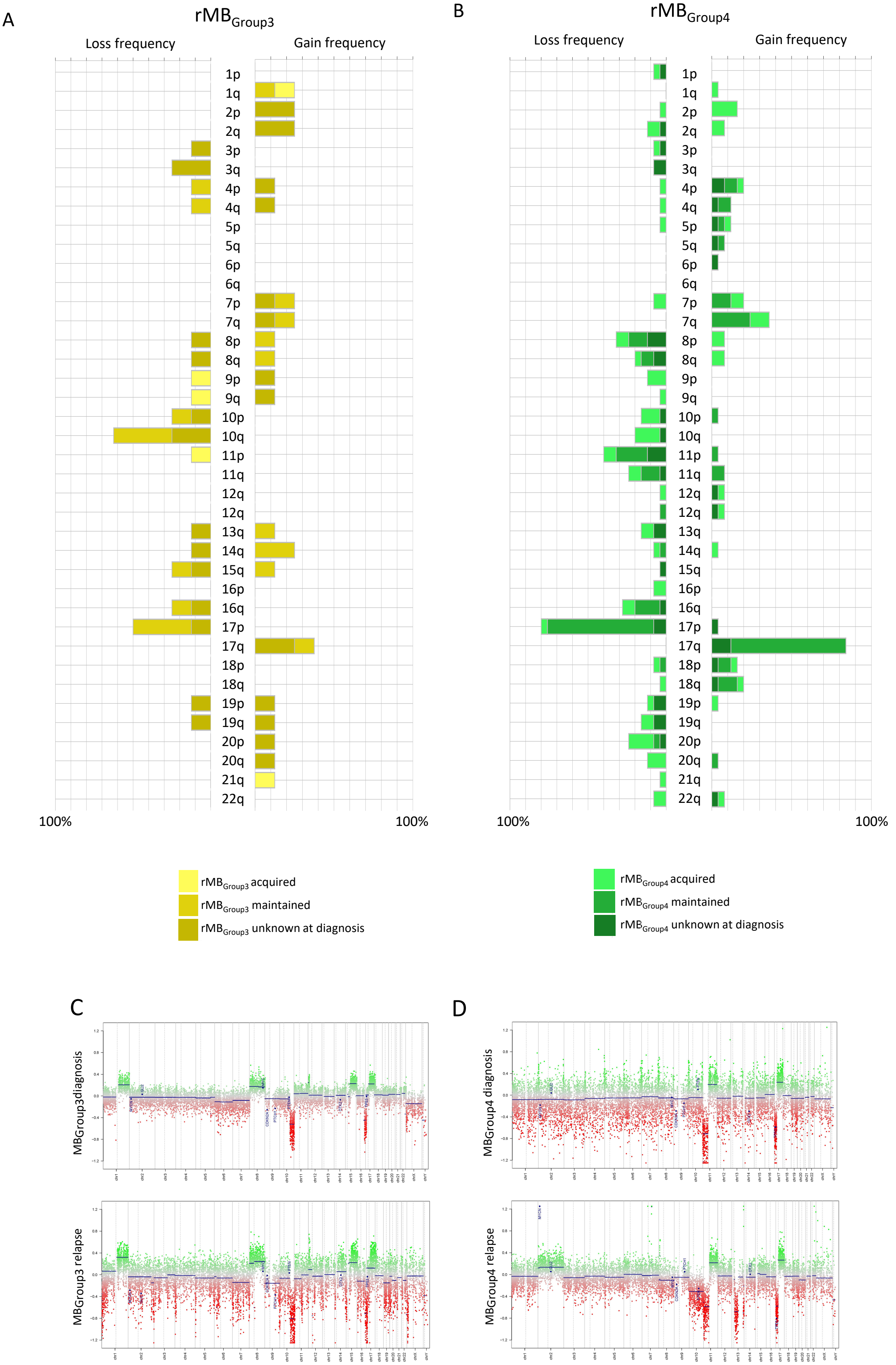

Supplementary Fig. 4. CNV in rMB_{Group3} and rMB_{Group4}.
(A) All chromosomal arm level CNVs in rMB_{Group3}. (B) All chromosomal arm level CNVs in rMB_{Group4}. (C) Conumee copy number trace of matched diagnostic and relapse MB_{Group3} showing maintenance of chromosome 8 gain and 10q loss amongst other CNVs. (D) Conumee copy number trace of matched diagnostic and relapse MB_{Group4} showing acquisition of focal *MYCN* amplification at relapse amongst other CNVs. CNV=copy number variation. rMB=relapse medulloblastoma.

Supplementary Fig. 5

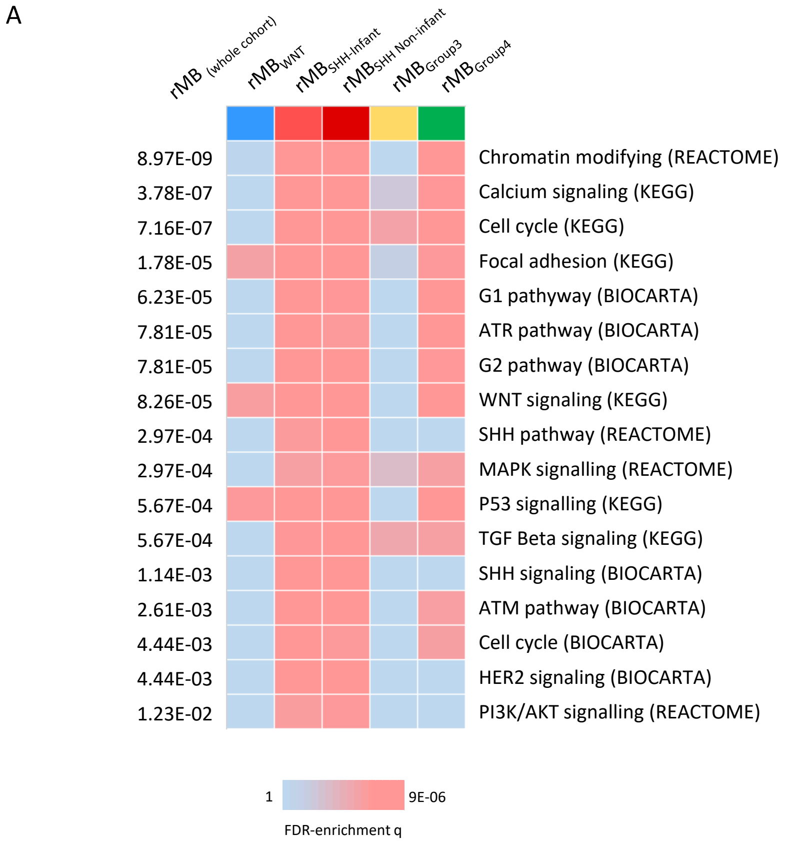

Supplementary Fig. 5. Gene set enrichment analysis of curated canonical pathways.
(A) Heatmap summarizing key canonical pathways from gene set enrichment analysis of rMB focal CNV and mutational gene sets. FDR- q values are reported for all rMBs and represented by subgroup in a heatmap with red indicating significant enrichment. rMB=relapsed medulloblastoma. WNT=wnt/wingless. SHH=sonic hedgehog. FDR q value= false discovery rate adjusted p value.
