# Supplementary material for: Emergence and maintenance of actionable genetic drivers at medulloblastoma relapse
Source: Neuro Oncol. 2021 Jul 17;24(1):153–65. doi: 10.1093/neuonc/noab178 (PMC8730763; doi:10.1093/neuonc/noab178)
Supplement: noab178_suppl_Supplementary_Methods [file noab178_suppl_supplementary_methods.docx]

#### Data availability

DNA methylation array data produced in this study have been deposited in ArrayExpress (<https://ebi.ac.uk/arrayexpress/>)(E-MTAB-9430, E-MTAB-9432). DNA sequencing data produced in this study have been deposited in the European Genome-phenome Archive and can be accessed with authorization from the Data Access Committee (https://ebi.ac.uk/ega/). Additional next generation sequencing datasets (EGAD00001000946) were obtained with authorization from the International Cancer Genome Consortium (<https://icgc.org>). Mutational data assembled for the independent control cohort were obtained from a published tumor study and corresponding DNA methylation array data was obtained upon request to the corresponding author (PMID: 28726821)^1^.

**Second generation molecular subtypes**

Second generation group 3 and group 4 molecular subtypes were assigned at diagnosis and relapse using DNA methylation-based classification ([www.](http://www/)molecularneuropathology.org/mnp) (confidence score >0.7)^2^ and confirmed using tSNE clustering of our cohort alongside published consensus group3 and group4 medulloblastoma subtype datasets^3^. Second generation MB_SHH_ subtypes were defined by tSNE clustering of our MB_SHH_ cohort alongside published MB_SHH_ subtype datasets^4^. Subtype calls were used to define clusters SHHα, SHHβ, SHHγ and SHHδ with the cluster centre defined as the average coordinate for each subtype using Euclidean distance. Distance from cluster centres was measured for each diagnosis and relapse sample and subtype assigned based on nearest cluster centre. Ratio to the second nearest cluster was calculated and samples with a ratio <0.7 were confidently assigned MB_SHH_ subtype. Where rMB tumors could be confidently assigned subtype rMB_SHH-Infant_ tumors consisted of SHHβ (n=4) and SHHγ (n=3) subtypes, and rMB_SHH Non-infant_ tumors consisted of SHHα (n=3) and SHHδ (n=14) subtypes.

#### Copy number analysis

Chromosomal arm-level and focal copy number changes (CNVs) were identified in Illumina DNA methylation array datasets (Illumina; San Diego, CA, USA)(relapsed MB and independent diagnostic MB cohorts) using the R package ‘conumee’ [(http://bioconductor.org/packages/conumee/](http://bioconductor.org/packages/conumee/))) as previously described^5^. Copy number profiles were generated from methylation array probe intensity values and focal CNV were called by interrogation of array probe log ratio within the genomic locations of candidate genes. Where methylation data was not available (rMB tumor samples obtained from ICGC NGS datasets), whole-exome sequencing datasets were analyzed for CNVs using Nexus Copy Number 10.0 (BioDiscovery; El Segundo, CA, USA) and the SNP- FASST2 segmentation algorithm employed to make copy number estimations. To confirm changes all focal CNVs identified were validated by visual inspection.

#### Mutational analysis

Whole-exome and targeted gene panel sequencing was performed using Agilent SureSelect target enrichment platform (Agilent Technologies; Santa Clara, CA, USA) and Illumina paired-end sequencing (Illumina; San Diego, CA, USA) according to manufacturer’s instructions. All next generation sequencing datasets were analyzed for coding/exonic region variants using Genome Analysis Toolkit (GATK) version 3.7, according to Broad Institute’s best practices (Burrows wheeler alignment, Haplotype Caller)^6^ and annotated using Ensembl Variant Effect Predictor^7^. Where germline data was available, only somatic variants were reported (except in cases of known germline predisposition gene mutations^8^).

**Supplementary references**

**1.** Northcott PA, Buchhalter I, Morrissy AS, et al. The whole-genome landscape of medulloblastoma subtypes. *Nature.* 2017; 547(7663):311-317.

**2.** Capper D, Jones DT, Sill M, et al. DNA methylation-based classification of central nervous system tumours. *Nature.* 2018; 555(7697):469-474.

**3.** Sharma T, Schwalbe EC, Williamson D, et al. Second-generation molecular subgrouping of medulloblastoma: an international meta-analysis of Group 3 and Group 4 subtypes. *Acta neuropathologica.* 2019; 138(2):309-326.

**4.** Cavalli FM, Remke M, Rampasek L, et al. Intertumoral heterogeneity within medulloblastoma subgroups. *Cancer cell.* 2017; 31(6):737-754. e736.

**5.** Schwalbe EC, Lindsey JC, Nakjang S, et al. Novel molecular subgroups for clinical classification and outcome prediction in childhood medulloblastoma: a cohort study. *The Lancet. Oncology.* 2017; 18(7):958-971.

**6.** Van der Auwera GA, Carneiro MO, Hartl C, et al. From FastQ data to high‐confidence variant calls: the genome analysis toolkit best practices pipeline. *Current protocols in bioinformatics.* 2013; 43(1):11.10. 11-11.10. 33.

**7.** McLaren W, Gil L, Hunt SE, et al. The ensembl variant effect predictor. *Genome biology.* 2016; 17(1):122.

**8.** Waszak SM, Northcott PA, Buchhalter I, et al. Spectrum and prevalence of genetic predisposition in medulloblastoma: a retrospective genetic study and prospective validation in a clinical trial cohort. *The Lancet Oncology.* 2018; 19(6):785-798.
